# Supplementary material for: A new look at weather-related health impacts through functional regression
Source: Sci Rep. 2018 Oct 15;8:15241. doi: 10.1038/s41598-018-33626-1 (PMC6189063; doi:10.1038/s41598-018-33626-1)
Supplement: Supplementary file 1 — Supplementary information [file 41598_2018_33626_MOESM1_ESM.pdf]

Supplementary information

A new look at weather-related health impacts through functional regression

Pierre Masselot[1*], Fateh Chebana[1], Taha B.M.J. Ouarda[1], Diane Bélanger[1,2], André St-Hilaire[1], Pierre Gosselin[1,2,3]

[1]Institut National de la Recherche Scientifique, Centre Eau-Terre-Environnement, Québec, Canada;

[2]Centre Hospitalier Universitaire de Québec, Centre de Recherche, Québec, Canada;

[3]Institut national de santé publique du Québec (INSPQ), Québec, Canada.

**Corresponding Author: pierre-lucas.masselot@ete.inrs.ca*

This appendix introduces mathematical details not covered in the paper's body. Among the details is the necessary preliminary step of producing functional data from the observations through smoothing. Then both the functional linear model for scalar response (SFLM) and the fully functional linear model (FFLM) are detailed.

1. Functional data

The starting point of FDA is to estimate the continuous function $x(t)$, $t \in T$ from the observations $x(t_l)$. The function $x(t)$ is obtained through data smoothing, by expressing it as a sum of basis functions, *i.e.*

$$\hat{x}(t) = \sum_{k=1}^{K} c_k \phi_k(t)\,, t \in T \tag{1}$$

where $\phi_k(t)$ is a set of analytically known basis functions (classically Fourier or B-spline bases, but other ones are possible), and c_k are the associated coefficients. Equation (1) allows the representation of a large variety of functions only through K scalar values c_k. Moreover, it allows controlling the smoothness of $\hat{x}(t)$ according to the value of K. The smaller K is, the smoother the estimated function $\hat{x}(t)$ is.

In practice, the coefficients c_k are estimated by minimizing the penalized sum of squares of errors (PSSE) criterion

$$PSSE = \sum_{l=1}^{L} \left(x(t_l) - \sum_{k=1}^{K} c_k \phi_k(t_l) \right)^2 + \lambda \int D^2 \hat{x}(t) dt \tag{2}$$

where the $x(t_l)$ are the measured data points at times t_l, $l = 1, \dots, L$ and λ is the parameter controlling the severity of the penalty $\int D^2 \hat{x}(t) dt$ with D^2 for the second derivative. This penalty represents the roughness of the resulting function $\hat{x}(t)$. The parameter λ is linked to the number of basis functions K: the higher λ is, the lower K is and conversely. λ is often chosen by minimizing a criterion such as cross-validation (Stone, 1974) or generalized cross-validation(Craven and Wahba, 1978). Note that one can also choose K subjectively, depending on the goal of the study (*e.g.* focusing on long-term variation). Finally, although Equations (1) and (2) express the estimation of a single curve, in practice the user has to estimate a set of N functional data $x_i(t)$, $i = 1, \dots, N$. In this case, the value of λ is chosen once and is the same for all N curves.

2. Functional linear models

Once the functional data have been obtained from discrete measurements, they can be used in a FLM. The model to use depends on the response variable: a) if it is intended as a discrete variable, then the SFLM has to be used, b) if the response can be expressed as a functional variable, it is better to use the FFLM. The difference between the two models is illustrated in Figure 2 of the main manuscript. Note that models with a functional response and a discrete exposure have also been extensively studied (Brumback and Rice, 1998) and can be of use in environmental epidemiology. However they are not considered in the present paper.

2.1. Functional linear model for scalar response

The SFLM is intended at estimating the effect of one or several functional exposures on a scalar response. For only one predictor, it is commonly expressed as (Hastie and Mallows, 1993):

$$y_i = \beta_0 + \int_T x_i(s)\beta_1(s)ds + \epsilon \quad (3)$$

where y_i is the discrete response, $x_i(s)$ is a functional exposure, β_0 is the intercept, $\beta_1(s)$ is the functional coefficient and ϵ is the residual error. The functional coefficient $\beta_1(s)$ gives the influence of the curve $x_i(s)$ at all times $s \in T$ on the response y_i. An example would be to express y_i the total mortality of year i according to the temperature curve $x_i(s)$ of the same year. In this case, β_0 is the mean annual mortality, $T = [0; 365]$ represents a year and $\beta_1(s)$ indicates the role played by temperature at time s of the year on the total mortality of the same year.

To estimate the model in Equation (3), the coefficient $\beta_1(s)$ is expressed as a sum of basis functions in the exact same way as the functional data in Equation (1), *i.e.* $\hat{\beta}_1(s) = \sum_{k=1}^{K_\beta} b_k\theta_k(s)$. Since the $\theta(s)$ functions are known, fitting model of Equation (3) reduces to estimating the parameters b_k. Therefore, the problem is reduced to a classical regression in a new space, which is the space of functions $\theta_k(s)$. This allows controlling for the complexity of $\hat{\beta}_1(s)$ by selecting the parameters b_k, usually by adding a penalty on the complexity of $\hat{\beta}_1(s)$ as in Equation (2) (Ramsay and Silverman, 2005). Note that the SFLM in Equation (3) can be more general by including several functional predictors and also non-functional covariates(*e.g.* Brockhaus et al., 2015). This could be useful for controlling unmeasured confounders.

Note that functional data analysis lacks asymptotic theory to compute parametric confidence intervals (CI) for $\hat{\beta}_1(t)$. Therefore in the present paper, CIs are computed through the non-parametric method of wild bootstrap (Gonzalez-Manteiga and Martinez-Calvo, 2011).

2.2. Fully functional linear model

The FFLM model aims at explaining a whole functional response $y_i(t)$, $t \in T$ according to a whole functional predictor $x_i(s)$, $s \in S$. Theoretically, T and S can be completely different, but we consider here $S = T$ both for simplicity and because it is effectively the case in the paper. The FFLM is expressed as:

$$y_i(t) = \beta_0(t) + \int_T x_i(s)\beta_1(s,t)ds + \epsilon(t)\,, t \in T \quad (4)$$

where $\beta_0(t)$ is the functional intercept (which can be interpreted as the mean function of the $y_i(t)$), $\beta_1(s,t)$ is the functional coefficient, and $\epsilon(t)$ the functional residual error (Ramsay and Silverman, 2005 chapter 16). Since both $y_i(t)$ and $x_i(s)$ are functional data, $\beta_1(s,t)$ is now a surface giving the influence of $x_i(s)$ on $y_i(t)$ at each possible couple s and t.

When T corresponds to a period of time, as it is often the case in environmental epidemiology, then the FFLM includes lags of the exposure, which is when $s < t$. However, the model in (4) also allows $\beta_1(s,t)$ to be non-null for $s > t$, which would mean that $x_i(s)$ influences $y_i(t)$ backwards in time. This is why (Malfait and Ramsay, 2003) proposed a particular case of the FFLM (called historical functional linear model, HFLM) taking only account of times $s \leq t$. Moreover, subsequent versions of the HFLM allow considering only a subset of times $s \in [l(t); u(t)]$ where $l(t) < u(t) \leq t$ (Brockhaus et al., 2016). Hence the HFLM is written as

$$y_i(t) = \beta_0(t) + \int_{l(t)}^{u(t)} x_i(s)\beta_1(s,t)ds + \epsilon(t), t \in T \quad (5)$$

For clarity purposes, the model in Equation (5) is also referred to as the FFLM in the paper, although it is the one effectively used in application 2.

To fit the FFLM in Equations (4) and (5), the coefficient surface $\beta_1(s,\mathrm{t})$ has to be expressed as a bivariate basis expansion (also called tensor product), *i.e.* $\hat{\beta}_1(s,t) = \sum_{k_1=1}^{K_1}\sum_{k_2=1}^{K_2} b_{k_1k_2}\theta_{k_1}(s)\theta_{k_2}(t)$. This time, the goal is to estimate the $K_1 \times K_2$ matrix containing all the $b_{k_1k_2}$ coefficients. The estimation involves Kronecker products and is more complex than for the SFLM. Therefore we refer to Ramsay and Silverman (2005) as well as Brockhaus et al. (2015) for details on how to estimate the matrix of coefficients for the model in Equation (4), and to Brockhaus et al. (2016) for the particular case of the model in Equation (5).

An important limitation of the method of Brockhaus et al. (2016) for the estimation of the HFLM is that there is no possibility of constraining the ends of the $\beta_1(s,t)$ surface to coincide to the same value. This is nonetheless important in applications involving cyclic processes. In the application discussed in the manuscript, this issue is addressed by extending the time period T by a month at each end. Therefore, in the application, for the estimation, the curves span the previous year December and next year January. This extra time is then removed from the estimated surface. Although this does not theoretically ensure that the surface is cyclic, in practice it reduces the boundary issues.

2.3. Coefficient estimation

Although the different FLMs have always been considered as different models, a recent work proposed a unified framework to estimate all FLMs (including the SFLM and FFLM) through the machine learning technique of boosting (Brockhaus et al., 2015, 2016). Boosting has intensively been used in big data applications since its introduction. The estimation procedure roughly consists in iteratively fitting a large number of simple models (usually called base-learners), *i.e.* with a small K_β in the case of the SFLM or small K_1 and K_2 in the case of the FFLM. All these simple models are averaged to obtain a powerful fit. The difference with classical ensemble estimation methods is that at each step, the new estimated base-learner focuses on fitting observations that were poorly fitted by the previous base-learners. Although this estimation method is the one used to produce the

results in the present work, the present section does not details the boosting algorithm, but we refer to Bühlmann and Hothorn (2007) for this.
